# Serological and Molecular Survey of Hepatitis E Virus in Small Ruminants from Central Portugal

**DOI:** 10.1007/s12560-024-09612-4

**Published:** 2024-09-05

**Authors:** Sérgio Santos-Silva, Jesús L. Romalde, Jaqueline T. Bento, Andreia V. S. Cruz, Pedro López-López, Helena M. R. Gonçalves, Wim H. M. Van der Poel, Maria S. J. Nascimento, António Rivero-Juarez, João R. Mesquita

**Affiliations:** 1 School of Medicine and Biomedical Sciences (ICBAS), University of Porto, Porto, Portugal; 2 Department of Microbiology and Parasitology, CIBUS-Faculty of Biology, Cross-Disciplinary Research Center in Environmental Technologies (CRETUS), Universidad de Santiago de Compostela, Santiago de Compostela, Spain; 3 University of Aveiro (UA), Aveiro, Portugal; 4 Unit of Infectious Diseases, Clinical Virology and Zoonoses, Hospital Universitario Reina Sofia, Instituto Maimonides de Investigación Biomédica de Córdoba (IMIBIC), Universidad de Córdoba (UCO), Cordoba, Spain; 5 Center for Biomedical Research Network (CIBER) in Infectious Diseases, Health Institute Carlos III, Madrid, Spain; 6 LAQV, REQUIMTE, Department of Chemistry and Biochemistry, Faculty of Sciences, University of Porto, Porto, Portugal; 7 Quantitative Veterinary Epidemiology, Wageningen University, Wageningen, The Netherlands; 8 Department Virology & Molecular Biology, Wageningen Bioveterinary Research, Lelystad, The Netherlands; 9 Faculty of Pharmacy, University of Porto (FFUP), Porto, Portugal; 10 Epidemiology Research Unit (EPIUnit), Instituto de Saúde Pública da Universidade Do Porto, Porto, Portugal; 11 Laboratory for Integrative and Translational Research in Population Health (ITR), Porto, Portugal

**Keywords:** HEV, One health, Small Ruminants, Zoonosis

## Abstract

Hepatitis E virus (HEV) is currently recognized as an emerging problem and a growing concern for public health in developed countries, with HEV infections mainly attributable to foodborne transmission of HEV-3. The zoonotic HEV genotype 3 infects a wide range of mammalian hosts, with swine considered as the primary host. This study investigates the occurrence of HEV among small ruminants in Portugal. The primary aim of the present research was to evaluate the circulation and the potential for HEV infection among sheep and goats. A total of 400 bile samples and 493 blood samples were collected from sheep and goats at a slaughterhouse in the center region of Portugal, between January 2022 and March 2023. The HEV RNA detection in bile samples was performed using a nested broad-spectrum RT-PCR targeting the ORF1 region. Serological analysis to detect anti-HEV antibodies was conducted using a commercial double-antigen sandwich multi-species ELISA. The HEV RNA was not detected in any bile samples using the nested broad-spectrum RT-PCR. Serological analysis revealed an overall HEV antibody seroprevalence of 2% (10/493, 95% CI: 0.98–3.70) among the small ruminants, namely 2.2% in goats and 2.0% in sheep. Curiously, no statistically significant association among the factors, age, sex and species and HEV seroprevalence was observed. Although HEV RNA was not detected in the bile of sheep and goats, this study the evidence of seroprevalence in these small ruminant species. Further research could provide additional insights into the factors influencing HEV transmission dynamics in small ruminants in Portugal and its potential implications for public health.

## Introduction

Hepatitis E virus (HEV) is currently recognized as an emerging problem and a growing concern for public health in developed countries where human infections are mainly due to a zoonotic foodborne transmission. The HEV is a single-stranded RNA virus with a genome length ranging from 6.4 to 7.3 kb and comprising three partially overlapping open reading frames (ORF1, ORF2, and ORF3). Viral particles exhibit a diameter ranging from 27 to 34 nm and are nonenveloped in feces and bile while in blood they circulate in a membrane-associated quasi-enveloped structure (Debing et al., 2016; Kamar et al., 2012; Nagashima et al., 2017; Takahashi et al., 2010). The HEV is a member of the family *Hepeviridae*, and subfamily *Orthohepevirinae* (Purdy et al., 2022), infecting mammals and birds. The subfamily *Orthohepevirinae* is further divided into four genera and among them, the *Paslahepevirus* genus contains the species *P. balayani*, which includes eight HEV genotypes (HEV-1 through HEV-8) that have the ability to infect humans and various other mammalian species. The HEV-1 and HEV-2 exclusively infect humans, while genotypes 3, 4, and 7 infect both humans and animals, and genotypes 5, 6, and 8 exclusively infect animals (Smith et al., 2020). The HEV stands out as the sole human hepatitis virus with documented zoonotic transmission among the five known hepatotropic viruses, and it ranks sixth among 887 wildlife viruses in terms of spillover potential (Grange et al., 2021).

In particular, the zoonotic HEV-3 and HEV-4 have swine as the primary hosts, being also detected in deer, ruminants, rabbits, dolphins, raccoons, lynxes, felines, canines, and equids (R. Ahmed & Nasheri, 2023). Human infections with HEV-3 and HEV-4 classically manifest as asymptomatic, with typical spontaneous resolution, obviating the need for therapeutic intervention (Hoofnagle et al., 2012). However, specific groups, such as immunocompromised individuals and those with preexisting chronic liver conditions, may experience a more severe course of the illness and encounter poorer outcomes (Kamar et al., 2012; Karna et al., 2020; Rivero-Juarez et al., 2019). Furthermore, it has been observed that HEV-3 infections can exhibit additional manifestations outside of the liver, with neurological symptoms being the most common (Cheung et al., 2012; Kamar et al., 2011). The primary route of transmission for HEV-3 is through the consumption of raw or undercooked pork meat (Velavan et al., 2021). Apart from the transmission route associated with the consumption of pork products, researchers are exploring alternative transmission routes, such as the consumption of milk derived from other species, including cows, sheep, goats, donkeys, and camels (King et al., 2018; Rodríguez-Lázaro et al., 2018; Santos-Silva et al., 2022; Treagus et al., 2021). As such, the routes for transmission of HEV-3 still remain to be fully understood (Pallerla et al., 2020; Takahashi et al., 2020). Recent investigations have revealed the presence of HEV-3 and HEV-4 in domestic small ruminants, suggesting that these animals could serve as significant reservoirs for these viruses (Di Martino et al., 2016; El-Mokhtar et al., 2020; Li et al., 2017; Long et al., 2017; Sarchese et al., 2019; Wu et al., 2015). Moreover, a previous study in Portugal has shown serological evidence of HEV circulation in sheep and has reported an increased risk of HEV infection in workers occupationally exposed to sheep (Mesquita et al., 2020).

Hepatitis E virus enters by the oral route, passing through the intestinal tract, where it replicates. The virus subsequently migrates to the liver, where it replicates before being released into the bile and bloodstream (Balayan et al., 1983; Capelli et al., 2019; Marion et al., 2020). Given the hepatotropic nature of HEV, some studies have demonstrated that liver samples may result in higher detection rates or viral loads compared to stool samples from the same animal specimen (Priemer et al., 2022). For instance, a study demonstrated that the HEV viral loads in stool of pigs were lower than those found in liver (García et al., 2019). Furthermore, a study investigating HEV RNA in sylvatic and captive wild boar from Portugal found higher detection rates in livers than in stools (Mesquita et al., 2016).

It is also crucial to recognize that a significant challenge in viral detection is frequently the limited efficiency of viral RNA extraction. As such, there is an inherent risk of false-negative detections due to inefficient RNA recoveries and for this reason internal controls of RNA extraction efficiency have been used (W. Ahmed et al., 2022). These challenges have also been previously noted to significantly contribute to the concerning situation where at least half of viral foodborne outbreaks remain undetected (Stals et al., 2012).

Considering the diagnostic challenges and focusing on bile to detect HEV RNA, the objective of this study was set to investigate the occurrence of HEV and other zoonotic hepevirus, such as *Rocahepevirus*, in the bile of small ruminants in Portugal, as well as to assess the circulation of HEV by examining anti-HEV antibodies in these animals.

## Materials and Methods

### Sample Collection

Small ruminants samples were obtained from a slaughterhouse of small ruminants located in the center region of Portugal (Serra da Estrela mountain range). This slaughterhouse slaughters approximately 42,000 animals every year, mainly juveniles. The geographical location of all sampled animals collected from the slaughterhouse were from the northern and central regions of Portugal.

A total of 400 bile samples and 493 blood samples were collected from both sheep and goats.

In January and March 2022, 400 bile samples (5 mL each) were collected from the biliary bladder on the slaughter line, following a modified method (Caravedo et al., 2021). These included 51 from adult sheep (≥ 1 year), 284 from lambs (3 months to 1 year), 18 from adult goats (≥ 1 year), and 47 from goatlings (3 months to 1 year).

From the 493 blood samples 354 were from sheep and 139 from goats, of which 95 were from adult sheep, 259 from lambs, 11 from adult goats and 128 from goatlings. Blood samples were collected in January 2022 and March 2023. A total of 184 blood samples were collected in January 2022. From those samples, 165 (156 from sheep and 9 from goats) were simultaneously collected with bile samples from the same individuals while from the remaining 19 individuals, only blood was possible to collect. Furthermore, the other 309 samples of blood were collected in March 2023 and this time no samples were simultaneously collected with bile samples. Blood was collected by placing a 5 mL sterilized tube upon exsanguination of each animal and serum was immediately separated by centrifugation at 400 g for 10 min. No animals were killed for the sake of this study.

All bile and serum samples were kept at 4 ºC and transported to the lab within 12 h. All samples were then stored at – 80 ºC until nucleic acid extraction from bile or serological analysis were performed.

### Nucleic Acid Extraction

Nucleic acid was extracted from 200 µL of each bile sample using the QIAamp Viral Mini Kit (Qiagen, Hilden, Germany), according to the manufacturer’s instructions, in the QIAcube® automated platform (Qiagen). Prior to extraction, 10 µL of the internal control mengovirus (MeV) clone vMC0 was added to the first sample of each extraction batch, with each batch consisting of 12 samples. The amount of the process control (MeV) added to each sample corresponded to about 10^3^ infective particles (Costafreda et al., 2006). Eluted RNA was stored at – 80 ºC with RNase-free water.

### Molecular Detection of HEV and MeV

To detect HEV RNA, a nested RT-PCR assay was used to amplify a 331–334 bp fragment of ORF1 region with the primer sets HEV-cs/HEV-cas for the first round and HEV-csn/HEV-casn for the second round (Johne et al., 2010). Oligonucleotides used for the molecular detection of HEV are shown in Table 1. All end-point PCR reactions were run on a T100 thermocycler (Bio-Rad). The reaction mixtures were performed using the Xpert One-Step RT-PCR kit (GriSP®, Porto, Portugal) for the first round and Xpert Fast Hotstart Mastermix 2 × with dye (GriSP®, Porto, Portugal) for the second round. The thermocycling conditions for the first round used were the following: cDNA synthesis at 45 ºC for 15 min, initial denaturation at 95 °C for 3 min, followed by 40 cycles of denaturation at 95 °C for 10 s, annealing at 50 ºC for 10 s, and extension at 72 °C for 15 s and a final extension at 72 °C for 10 min. For the second round an initial denaturation at 95 ºC for 3 min was performed, followed by 40 cycles of denaturation at 95 °C for 15 s, annealing at 50 ºC for 15 s, and extension at 72 °C for 2 s and a final extension at 72 °C for 10 min. The amplified DNA fragments were identified through electrophoresis on one percent agarose gel, which were subsequently stained with Xpert Green Safe DNA gel dye to visualize the PCR amplification products (GriSP®, Porto, Portugal) at a voltage of 120 V for 30 min. The UV transilluminator was used to validate and verify the obtained results.

**Table 1:** Oligonucleotides used for the molecular identification and/or characterization of HEV and MeV

Target organism	Oligonucleotide	Sequence (5´–3´)	Reference
HEV	HEV-cs	TCGCGCATCACMTTYTTCCARAA	(Johne et al., 2010)
	HEV-cas	GCCATGTTCCAGACDGTRTTCCA	
	HEV-csn	TGTGCTCTGTTTGGCCCNTGGTTYCDG	
	HEV-casn	CCAGGCTCACCRGARTGYTTCTTCCA	
MeV	Mengo110	GCGGGTCCTGCCGAAAGT	(Pintó et al., 2009)
	Mengo209	GAAGTAACATATAGACAGACGCACAC	
	Mengo147	Cy5-ATCACATTACTGGCCGAAGC-MGB NFQ	

For MeV detection, a quantitative RT-PCR (RT-qPCR) assay was used. Oligonucleotides used for the molecular detection of MeV are shown in Table 1. Reactions were run on a CFX Connect Real-Time PCR Detection System (Bio-Rad, Hercules, CA, USA) with the Xpert One-Step Fast Probe (GRiSP®, Porto, Portugal). The thermal cycling conditions for the RT-qPCR reaction included initial reverse transcription (RT) at 50 °C for 15 min, followed by a simultaneous step for reverse transcriptase inactivation and the initial denaturation of cDNA at 95 °C for 5 min. Consequently, 40 cycles of amplification were carried out, involving denaturation at 95 °C for 5 s and annealing/extension at 60 °C for 20 s. Afterwards, results were analyzed using the CFX Maestro 1.0 Software version 4.0.2325.0418 (Bio-Rad, Hercules, CA, USA).

### Calculation of Extraction efficiency

The MeV internal control's viral extraction effectiveness or recovery rate was estimated by comparing the threshold cycle (Ct) value for positive amplification of the MeV control with the Ct value for a sample, and it was classified as unacceptable (< 1%), acceptable (1–10%), and good (> 10%) (da Silva et al., 2007).

### Detection of anti-HEV antibodies

A commercial multi-species ELISA kit (HEV 4.0v; MP Diagnostics, Illkirch, France) was utilized to assess the presence of total anti-HEV antibodies, in accordance with the manufacturer's instructions. The assay relies on a recombinant protein ET2.1 derived from the highly conserved HEV capsid (Hu et al., 2008). The assay has a reported sensitivity of 99.2% and specificity of 99.2%.

### Statistical Analysis

The occurrences of HEV were calculated by dividing the number of positive animals by the total number of animals tested. This calculation was done using two-sided exact binomial 95% confidence intervals (95% CI). In order to investigate the association between the presence of anti-HEV antibodies and demographic factors such as age and gender, statistical analysis was performed using GraphPad Prism 5.0 software (GraphPad Software, Inc., San Diego, CA, USA), employing either the Chi-square (χ2) or Fisher's exact test. A significance level of *p* < 0.05 was utilized to determine the statistical significance of observed differences.

## Results

The HEV RNA was not detected in any of the bile samples of the 400 individuals tested. The nucleic acid extraction efficiency of the bile samples was deemed satisfactory, with MeV recovery rates ranging from 20.09 to 70.55%, and an average of 45.13%.

As for the serological analysis, an overall prevalence of antibodies anti-HEV of 2% (10/493; 95% CI: 0.98–3.70) was found in these two species of small ruminants. The seroprevalence of HEV between sheep and goat did not vary significantly, although the seroprevalence was slightly higher in goat than sheep (Table 2). The prevalence of anti-HEV antibodies was higher in adults than in juveniles animals in both species, but the comparison of seroprevalence between adult sheep (4.2%) and lamb (1.2%) or adult goat (9.1%) and goatling (1.6%) showed no statistically significant difference (*p* = 0.087 and *p* = 0.221, respectively) (Table 3 and 4). No significant difference was observed in HEV seroprevalence regarding the sex of the animals, either in sheep (*p* = 0.106) or in goats (*p* = 0.205). Seropositive goats and sheep were found in both sampling years, 2022 and 2023.

**Table 2:** Comparative seroprevalence analysis of anti-HEV antibodies in goats and sheep from the slaughterhouse

Variable	Categories	No. Positives/ no. Analysed	Seroprevalence (%) (95% CI)	*p*
Species	Sheep	7/354	2.0 (0.8–4.0)	1.000
	Goat	3/139	2.2 (0.5–6.2)

**Table 3:** Comparative seroprevalence analysis of anti-HEV antibodies in sheep by age and sex

Variable	Categories	No. Positives/ no. Analyzed	Seroprevalence (%) (95% CI)	*p*
Age	Lamb	3/259	1.2 (0.2–3.4)	0.087
	Adult	4/95	4.2 (1.2–10.4)	
Sex*	Female	3/143	2.1 (0.4–6.0)	0.106
	Male	1/4	25 (0.6–80.6)	
Sampling date	January/22	4/174	2.3 (0.6–5.8)	0.720
	March/23	3/180	1.7 (0.3–4.8)	

^*^The sex of some animals could not be recorded due to a field error

**Table 4:** Comparative seroprevalence analysis of anti-HEV antibodies in goats by age and sex

Variable	Categories	No. Positives/ no. Analyzed	Seroprevalence (%) (95% CI)	*p*
Age	Goatling	2/128	1.6 (0.2–5.5)	0.221
	Adult	1/11	9.1 (0.2–41.3)	
Sex*	Female	2/27	7.4 (0.9–24.3)	0.205
	Male	0/32	–	
Sampling date	January/22	1/10	10 (0.3–44.5)	0.202
	March/23	2/129	1.6 (0.2–5.5)	

^*^The sex of some animals could not be recorded due to a field error

## Discussion

The presence of HEV RNA in sheep and goats has been reported in several regions of the world, including in Europe (Di Martino et al., 2016; El-Mokhtar et al., 2020; Sarchese et al., 2019; Velavan et al., 2021; Wu et al., 2015). The only HEV genotype identified so far in small ruminants in Europe, more specifically in Italy, has been genotype 3, the most well-known zoonotic genotype (Di Martino et al., 2016; Sarchese et al., 2019; Velavan et al., 2021).

To date, in Portugal, only one study has shown serological evidence of HEV circulation in small ruminants, namely sheep (Mesquita et al., 2020). In the present study, we further used molecular and serological methods to search for the presence of HEV in sheep and goat from Portugal.

Here we present the first data from Portugal regarding the molecular screening of HEV RNA in bile samples from sheep and goats. As HEV replicates in the intestinal tract and migrates to the liver, where it replicates before being released into the bile (Yadav & Kenney, 2021), our investigation focused on bile collected in the gallbladder to identify HEV RNA. The success of using bile to detect HEV in pigs was demonstrated in a study from Japan (Uema et al., 2022), prompting us to undertake a similar investigation with the objective of detecting HEV presence in small ruminants in Portugal. Furthermore, the widespread circulation of HEV-3 in domestic and wild animals has been already demonstrated in Portugal (Berto et al., 2012; Moraes et al., 2022; Santos-Silva et al., 2023), lead us to think that the circulation of HEV in small ruminant species could be a possibility as a result of the frequent interspecies interactions.

In the present study, no HEV RNA was detected in any of the screened bile samples. The potential risk of being false-negative test due to an inefficient RNA recovery from bile samples was discarded given the high recovery rates (average 45.13%) of MeV used as the internal control of RNA extraction efficiency. Moreover, the nested broad-spectrum RT-PCR assay used has previously demonstrated high sensitivity and specificity, and it has been shown to detect known and novel HEV strains (Johne et al., 2010).

To date, several studies have focused on the search of HEV RNA in serum, stool, rectal swabs and blood of sheep and goats, with some reporting the absence of HEV RNA (Caballero-Gómez et al., 2022; Sanford et al., 2013; Tritz et al., 2018), while others have shown the presence of it with detection rates ranging between 0.15% and 100% in small ruminants (Batmagnai et al., 2023; Di Martino et al., 2016; Dziedzinska et al., 2020; El-Mokhtar et al., 2020; Geng et al., 2010; Li et al., 2017; Long et al., 2017; Palombieri et al., 2020; Sarchese et al., 2019; Wu et al., 2015; Yu et al., 2009).The HEV molecular characterization performed in some of these studies identified the following genotypes and subgenotypes, HEV-3 (Di Martino et al., 2016), HEV-3a (El-Mokhtar et al., 2020), HEV-3c (Sarchese et al., 2019), HEV-4 (Batmagnai et al., 2023), HEV-4d (Wu et al., 2015) and HEV-4h (Li et al., 2017; Long et al., 2017). Although in our study no HEV RNA was detected in bile samples, comparison with studies reporting the presence of this marker of active infection should be taken with care since they used different sample sizes and tested different type of samples.

To date HEV-4 was reported only in small ruminants in China (Huang et al., 2016; Li et al., 2017; Long et al., 2017; Wu et al., 2015), and in sheep from Mongolia (Batmagnai et al., 2023). In Europe only HEV-3 has been detected in small ruminants (Di Martino et al., 2016; Sarchese et al., 2019). Similarly, in Egypt, HEV-3a was identified in goat samples (El-Mokhtar et al., 2020). In China, HEV-4, subtype 4d and 4 h, have been also detected in cows and in Yellow cattle (Huang et al., 2016; Yan et al., 2016). All together these findings highlight the vast genetic diversity of HEV circulating in ruminants.

Although, the present study lacked molecular confirmation of HEV in bile samples, anti-HEV antibodies were found, demonstrating that these small ruminant species are susceptible to HEV infection. Interestingly, there was no significant statistical difference of HEV seroprevalence between sheep and goats, juveniles and adult animals, gender and sampling date. In spite of this, the contact between farmed small ruminants and the HEV primary reservoir pig, should be considered as a potential explanation for interspecies transmission as well as their contact with the farmer or their contaminated workwear, as shown in a previous study from the region (Mesquita et al., 2020). This underscores the importance of considering various pathways for virus transmission and implementation of appropriate preventive measures to mitigate the spread of HEV within agricultural settings.

While our study provides important insights, it is worth noting that samples were collected from a slaughterhouse setting, which limited the scope of farm-level data collection. For more detailed epidemiological analysis, future research would benefit from direct farm sampling to provide a more comprehensive view of HEV prevalence and transmission patterns.

In Europe, some studies have been performed to assess the seroprevalence of HEV in small ruminant species. In Bulgaria, a HEV seropositivity of 32.2% and 24.4% were found in sheep and goats, respectively (Tsachev et al., 2023). In Spain, two serological surveys have also detected anti-HEV antibodies in small ruminant, having the study performed in the Catalonia region, found a seroprevalences of 1.92% in sheep and 0.60% in goats, using a commercial genotype 3-based ELISA (Peralta et al., 2009). Another study from southern Spain reported seroprevalences of 2.1% in sheep and 13.8% in goats (Caballero-Gómez et al., 2022) and identified goats and the overall small ruminant population on the farm as potential risk factors for HEV exposure. Moreover, the study revealed that the seropositivity of farmed goats and the number of small ruminants in the farm were linked to an HEV exposure among small ruminants in the region (Caballero-Gómez et al., 2022). In Africa, a study conducted in Burkina Faso reported a HEV seroprevalence of 12% in sheep and 28.4% in goats using a multi-species ELISA (Ouoba et al., 2019). In Portugal, only one HEV serological study has been conducted in small ruminants so far, namely in sheep where a seroprevalence of 16.6% was detected (Mesquita et al., 2020). Curiously, in the present study we found a much lower HEV seroprevalence (2.0%) in sheep from the same region of Portugal, but a different commercial ELISA was used. This discrepancy could be attributed to the comparative sensitivity and specificity of the kits employed, as the MP Bio HEV ELISA 4.0v used in our study has a high sensitivity (97.1%) and specificity (99.1%), which might differ slightly from other commercial ELISA kits previously used in the region. The diversity of seroprevalences reported in sheep and goats so far must be interpreted carefully as different sample size and different ELISA assays were used in all these studies which highlights the need for continued monitoring of the seroprevalence of HEV in small ruminants in the region.

Furthermore, virus shedding is a concerning factor in virus transmission among swine farms (Kanai et al., 2010). The same scenario could arise in small ruminant farms, underscoring the significance of stringent sanitary measures for maintaining healthy livestock management in the country. Moreover, since HEV has been found in small ruminant liver (Li et al., 2017) careful handling by workers in slaughterhouses and meat processing facilities is required.

In summary, while the current study did not detect HEV RNA in the bile of sheep and goats, it does provide evidence of HEV infection through the prevalence of anti-HEV antibodies in these small ruminants. Consequently, further investigation is warranted, emphasizing the necessity for comprehensive epidemiological surveys and improved communication regarding HEV infection risk factors in these species. Future research should consider direct sampling at farm levels to gather more comprehensive data, which will enhance understanding of HEV transmission dynamics and contribute to the development of targeted control measures.

## Data Availability

The data that support the findings of this study are available from the corresponding author upon reasonable request.
